# Use of FGF21 analogs for the treatment of metabolic disorders: a systematic review and meta-analysis

**DOI:** 10.20945/2359-4292-2022-0493

**Published:** 2023-11-10

**Authors:** Maria Paula Carbonetti, Fernanda Almeida-Oliveira, David Majerowicz

**Affiliations:** 1 Universidade Federal do Rio de Janeiro Faculdade de Farmácia Rio de Janeiro RJ Brasil Faculdade de Farmácia, Universidade Federal do Rio de Janeiro, Rio de Janeiro, RJ, Brasil; 2 Universidade Federal do Rio de Janeiro Instituto de Bioquímica Médica Leopoldo de Meis Rio de Janeiro RJ Brasil Instituto de Bioquímica Médica Leopoldo de Meis, Universidade Federal do Rio de Janeiro, Rio de Janeiro, RJ, Brasil; 3 Universidade do Estado do Rio de Janeiro Rio de Janeiro RJ Brasil Programa de Pós-graduação em Biociências, Universidade do Estado do Rio de Janeiro, Rio de Janeiro, RJ, Brasil

**Keywords:** FGF21, blood glucose, metabolic syndrome, glycated hemoglobin A, fasting, blood pressure, obesity, lipids

## Abstract

FGF21 is a hormone produced primarily by the liver with several metabolic functions, such as induction of heat production, control of glucose homeostasis, and regulation of blood lipid levels. Due to these actions, several laboratories have developed FGF21 analogs to treat patients with metabolic disorders such as obesity and diabetes. Here, we performed a systematic review and meta-analysis of randomized controlled trials that used FGF21 analogs and analyzed metabolic outcomes. Our search yielded 236 articles, and we included eight randomized clinical trials in the meta-analysis. The use of FGF21 analogs exhibited no effect on fasting blood glucose, glycated hemoglobin, HOMA index, blood free fatty acids or systolic blood pressure. However, the treatment significantly reduced fasting insulinemia, body weight and total cholesterolemia. None of the included studies were at high risk of bias. The quality of the evidence ranged from moderate to very low, especially due to imprecision and indirection issues. These results indicate that FGF21 analogs can potentially treat metabolic syndrome. However, more clinical trials are needed to increase the quality of evidence and confirm the effects seen thus far.

## INTRODUCTION

Discovered in 2000, fibroblast growth factor 21 (FGF21) is a peptide hormone of the endocrine FGF subfamily, along with FGF23 and FGF15/19 (1). The liver is the primary site of the production of this hormone, although white and brown adipose tissues (WAT and BAT) also express this gene (1–3). The structural domains of FGFs are well conserved, and the core region of the protein consists of 12 β-sheets. However, FGF21, similar to other FGFs of the endocrine subfamily, lacks a heparin-binding site. This difference means that these FGFs do not bind to endothelial receptors and can travel greater distances through the bloodstream (4). To act on target cells, FGF21 binds to FGF receptors and β-klotho coreceptors, which activate the extracellular signal-regulated kinase pathway. However, how FGF21 causes its multiple cellular effects remains unclear (5). Dipeptidyl peptidase 4 and fibroblast activation protein (FAP) can cleave the N- and C-terminal regions of FGF21. C-terminal cleavage by FAP shortens the hormone's half-life in circulation (6).

Various factors, such as diet, exercise, drug use, and metabolic conditions, regulate the gene expression and levels of FGF21 (6,7). Fasting and specific diets (ketogenic, low-calorie, and methionine-restricted) increase plasma levels and gene expression of FGF21 in both animals and humans (8–12). High-fat and sucrose-rich diets also increase plasma levels and gene expression of FGF21 in the liver and pancreatic islets (13–16). In cell culture, high concentrations of glucose and fatty acids increase FGF21 in pancreatic islets and hepatocytes (13,17). In addition, fatty acid infusion increases plasma levels of FGF21 (17).

Using overexpression or knockout models allows us to investigate the metabolic effects of FGF21. First, FGF21 knockout mice exhibit increased glucose tolerance (18). Moreover, knockout or knockdown pancreatic β-cells are less autophagic (13). On the other hand, mice overexpressing FGF21 exhibit reduced body weight, with reduced blood glucose, insulin, total cholesterol, and triglycerides (TAG). Furthermore, increased expression of FGF21 increases the concentration of bile salts in the liver and small intestine, probably caused by significant changes in the expression profile of genes in the metabolism of cholesterol and bile salts in these organs. These models also show reduced hepatic TAG and cholesterol in the stool (19).

The functions of FGF21 have spurred several research groups to test the effects of treatment with FGF21 or developed analogs on models of metabolic diseases such as obesity and diabetes (20). In preclinical trials, using FGF21 reduced weight gain and fat and lean mass, independent of food and water consumption, which increased in some studies (21–25). Increased energy expenditure and thermogenesis help explain these effects (26,27). Regarding plasma concentrations of energy substrates and other metabolic substances, treatment with FGF21 reduced glucose and glycated hemoglobin, TAG, and total cholesterol, with a reduction in VLDL and LDL but increased levels of HDL and plasma fatty acids (21,25,28–31). Furthermore, using FGF21 analogs increased plasma levels of β-hydroxybutyrate, indicating induction of hepatic β-oxidation (26,28).

Different factors help to explain this improvement in glucose homeostasis. First, FGF21 increases glucose tolerance and insulin sensitivity, reducing the HOMA index and increasing phosphorylation of protein kinase B and extracellular signal-regulated kinase in WAT and BAT. In addition, FGF21 reduces glucose production in the liver, inhibiting the expression of glucose-6-phosphate phosphatase (G6Pase) and phosphoenolpyruvate carboxykinase (PEPCK). Finally, FGF21 increases tissue glucose uptake, stimulating the expression of the GLUT1 glucose transporter, and increases hepatic glycogen synthesis (28,31–35). The improvement in blood lipid levels corroborates the effect of FGF21 on the reduction of atheroma plaque in mice on a high-fat diet (32).

Regarding the cardiovascular system, treatment with FGF21 analogs increases heart rate and blood pressure but improves heart condition and endothelial function (36–38). This treatment reduces inflammation in the blood vessels and the amount of cholesterol in the arteries (39). FGF21 also regulates the levels of various hormones in the blood, reducing insulin, leptin, and glucagon levels while increasing the concentration of adiponectin and FGF21 itself (21,28,32). In this sense, treatment with FGF21 analogs increases the number of pancreatic islets and insulin secretion (40). In addition, FGF21 acted on the liver of the models, reducing organ weight, in addition to the amount of TAG and cholesterol, hepatic steatosis scores, plasma levels of hepatic enzymes, and the expression of inflammatory and fibrotic genes, indicating a reduction in liver damage (21,24,41,42). FGF21 increases cholesterol concentration in the feces and reduces lipid synthesis in the liver through sterol-responsive element-binding protein 2 (SREBP-2) inhibition (39). These effects help explain the reduction in cholesterolemia and the levels of hepatic cholesterol.

In adipose tissue, treatment with FGF21 analogs increases the expression of GLUT1 and peroxisome proliferator-activated receptor γ coactivator 1α in WAT, indicating a greater capacity for glucose uptake and mitochondrial respiration. In BAT, FGF21 also alters the gene expression profile, leaving cells with greater thermogenic, glucose uptake, lipogenic, and lipolytic capacity (23,28,31,43).

FGF21 also acts on the immune and inflammatory system, reducing *ex vivo* secretion of IL-1β by human macrophages and reducing the expression of MMP-9 and ICAM-1 in WAT, in addition to the amount of CD68 cells in this tissue (9,28,32). In addition, using FGF21 reduces TNF-α levels (37).

In diabetic or obese models, FGF21 analog treatment reduced urine volume, the amount of creatinine in the urine, and the plasma levels of urea and creatinine (22,30). The same treatment increased the amount of chlorine in the urine (22). Furthermore, using FGF21 analogs reduces the amount of TAG and cholesterol in the kidneys, the levels of lipid peroxidation, and inflammatory and fibrotic factors in the kidneys (30). These results indicate an improvement in diabetes and kidney function.

In bone metabolism, using FGF21 increases plasma levels of CTX-1, indicating induction of bone resorption (25). These results indicate that FGF21-based therapies are promising for the treatment of nonalcoholic fatty liver disease, type II diabetes, and obesity (44,45).

The positive results of FGF21 analogs in preclinical trials stimulated the pharmaceutical industry to move toward clinical trials. Thus, in phase I and II clinical trials, several companies have tested FGF21 analogs created with different technologies in healthy humans or those with metabolic disorders. Here, we performed a systematic review and meta-analysis of clinical trials using FGF21 analogs to summarize the evidence of success or failure of this type of treatment for the future.

## MATERIALS AND METHODS

### Search for articles and inclusion criteria

We followed the PRISMA 2020 guide (46) to develop this systematic review and meta-analysis (Table S1). We searched for articles indexed in the PubMed, Scopus, and SciELO databases up to March 2023 using the search key ((((men) OR (women)) AND (obesity) OR (diabetes) OR (dyslipidemia) OR (hypertension)) AND (FGF21 AND (agonist OR analog))) AND (glycemia OR hb1ac OR HOMA OR weight OR cholesterol OR FFA OR “blood pressure”). The three authors independently analyzed the abstracts of articles obtained in the search, and we included only randomized clinical trials of FGF21 analogs versus placebos. In addition, the three authors independently analyzed the included articles and excluded articles that did not have extractable data from the studied outcomes, namely, fasting blood glucose, glycated hemoglobin, fasting blood insulin, HOMA index, body weight, total blood cholesterol, systolic blood pressure, and blood free fatty acids.

**Table S1:** PRISMA checklist

Section and Topic	Item #	Checklist item	Location where item is reported
**TITLE**			
Title	1	Identify the report as a systematic review.	Page 1
**ABSTRACT**			
Abstract	2	See the PRISMA 2020 for Abstracts checklist.	Page 1
**INTRODUCTION**			
Rationale	3	Describe the rationale for the review in the context of existing knowledge.	Pages 1 and 2
Objectives	4	Provide an explicit statement of the objective(s) or question(s) the review addresses.	Page 2
METHODS			
Eligibility criteria	5	Specify the inclusion and exclusion criteria for the review and how studies were grouped for the syntheses.	Page 3
Information sources	6	Specify all databases, registers, websites, organizations, reference lists and other sources searched or consulted to identify studies. Specify the date when each source was last searched or consulted.	Page 3
Search strategy	7	Present the full search strategies for all databases, registers and websites, including any filters and limits used.	Page 3
Selection process	8	Specify the methods used to decide whether a study met the inclusion criteria of the review, including how many reviewers screened each record and each report retrieved, whether they worked independently, and if applicable, details of automation tools used in the process.	Page 3
Data collection process	9	Specify the methods used to collect data from reports, including how many reviewers collected data from each report, whether they worked independently, any processes for obtaining or confirming data from study investigators, and if applicable, details of automation tools used in the process.	Page 3
Data items	10a	List and define all outcomes for which data were sought. Specify whether all results that were compatible with each outcome domain in each study were sought (e.g., for all measures, time points, analyses), and if not, the methods used to decide which results to collect.	Page 3
	10b	List and define all other variables for which data were sought (e.g., participant and intervention characteristics, funding sources). Describe any assumptions made about any missing or unclear information.	Page 3
Study risk of bias assessment	11	Specify the methods used to assess risk of bias in the included studies, including details of the tool(s) used, how many reviewers assessed each study and whether they worked independently, and if applicable, details of automation tools used in the process.	Page 3
Effect measures	12	Specify for each outcome the effect measure(s) (e.g., risk ratio, mean difference) used in the synthesis or presentation of results.	Page 3
Synthesis methods	13a	Describe the processes used to decide which studies were eligible for each synthesis (e.g., tabulating the study intervention characteristics and comparing against the planned groups for each synthesis (item #5)).	Page 3
	13b	Describe any methods required to prepare the data for presentation or synthesis, such as handling of missing summary statistics or data conversions.	Page 3
	13c	Describe any methods used to tabulate or visually display results of individual studies and syntheses.	Page 3
	13d	Describe any methods used to synthesize results and provide a rationale for the choice(s). If meta-analysis was performed, describe the model(s), method(s) to identify the presence and extent of statistical heterogeneity, and software package(s) used.	Page 3
	13e	Describe any methods used to explore possible causes of heterogeneity among study results (e.g., subgroup analysis, meta-regression).	Page 3
	13f	Describe any sensitivity analyses conducted to assess robustness of the synthesized results.	Page 3
Reporting bias assessment	14	Describe any methods used to assess risk of bias due to missing results in a synthesis (arising from reporting biases).	Page 3
Certainty assessment	15	Describe any methods used to assess certainty (or confidence) in the body of evidence for an outcome.	Page 3
**RESULTS**			
Study selection	16a	Describe the results of the search and selection process, from the number of records identified in the search to the number of studies included in the review, ideally using a flow diagram.	Fig. S1
	16b	Cite studies that might appear to meet the inclusion criteria, but which were excluded, and explain why they were excluded.	Page 3
Study characteristics	17	Cite each included study and present its characteristics.	Table S2
Risk of bias in studies	18	Present assessments of risk of bias for each included study.	Fig. S2
Results of individual studies	19	For all outcomes, present, for each study: (a) summary statistics for each group (where appropriate) and (b) an effect estimate and its precision (e.g., confidence/credible interval), ideally using structured tables or plots.	Pages 5 and 8
Results of syntheses	20a	For each synthesis, briefly summarize the characteristics and risk of bias among contributing studies.	Pages 5 and 8
	20b	Present results of all statistical syntheses conducted. If meta-analysis was performed, present for each the summary estimate and its precision (e.g., confidence/credible interval) and measures of statistical heterogeneity. If comparing groups, describe the direction of the effect.	Pages 5 and 8
	20c	Present results of all investigations of possible causes of heterogeneity among study results.	Pages 5 and 8
	20d	Present results of all sensitivity analyses conducted to assess the robustness of the synthesized results.	Pages 5 and 8
Reporting biases	21	Present assessments of risk of bias due to missing results (arising from reporting biases) for each synthesis assessed.	Fig. S2
Certainty of evidence	22	Present assessments of certainty (or confidence) in the body of evidence for each outcome assessed.	Table 1
**DISCUSSION**			
Discussion	23a	Provide a general interpretation of the results in the context of other evidence.	Pages 9, 10, and 12
	23b	Discuss any limitations of the evidence included in the review.	Page 12
	23c	Discuss any limitations of the review processes used.	Page 12
	23d	Discuss implications of the results for practice, policy, and future research.	Page 12
**OTHER INFORMATION**			
Registration and protocol	24a	Provide registration information for the review, including register name and registration number, or state that the review was not registered.	Page 12
	24b	Indicate where the review protocol can be accessed, or state that a protocol was not prepared.	Page 12
	24c	Describe and explain any amendments to information provided at registration or in the protocol.	Page 12
Support	25	Describe sources of financial or nonfinancial support for the review, and the role of the funders or sponsors in the review.	Page 12
Competing interests	26	Declare any competing interests of review authors.	Page 12
Availability of data, code and other materials	27	Report which of the following are publicly available and where they can be found: template data collection forms; data extracted from included studies; data used for all analyses; analytic code; any other materials used in the review.	Page 12

### Data extraction and analysis

The three authors independently extracted data from the included articles. First, we extracted the data manually, or when the manuscript presented the data in the form of graphs, we used the program WebPlotDigitizer 4.5 (47). Finally, we analyzed the data obtained using RevMan 5.4.1 software with random effects and standard mean difference methods.

### Analysis of risk of bias and summary of the quality of evidence

First, two authors independently analyzed the included articles for risk of bias using the RoB 2 algorithm (48). Then, we assessed the risk of publication bias based on the funnel plots generated by RevMan 5.4.1 and using Egger's test (49). Finally, we summarize the evidence-based quality on the GRADE algorithm (50).

## RESULTS

### Systematic review

**Figure S1:**
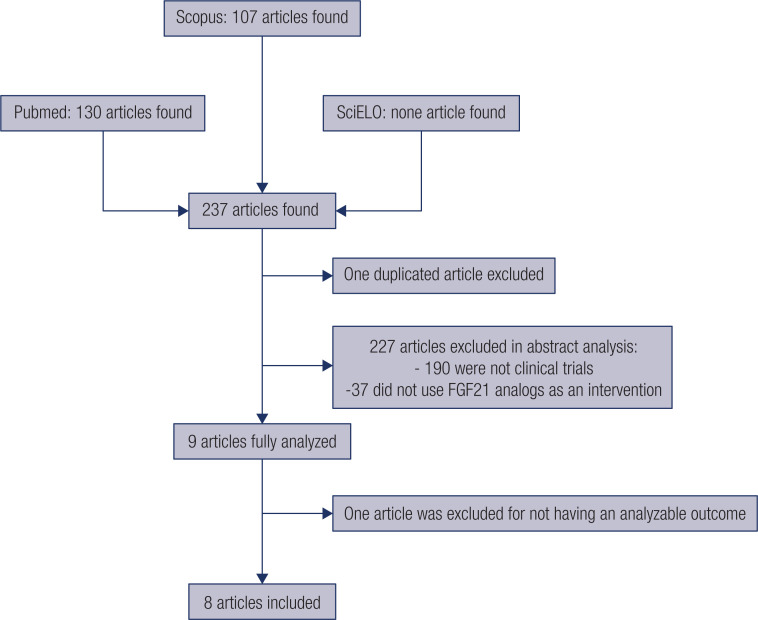
Flow chart of article selection for meta-analysis.

Our search yielded 237 published articles, one of which was duplicated between databases. We excluded 227 during abstract analysis: 190 were not clinical trials, and 37 did not use FGF21 analogs as an intervention. Thus, we thoroughly analyzed nine articles in total. We excluded one of them (51) for not having an analyzable outcome. Thus, we included eight randomized clinical trials (52–58) in the meta-analysis (Figure S1). Table S2 contains a summary of the characteristics of each study and the participants included in this meta-analysis. The studies included participants with an average age of 54, mostly men (59%) and white (87%). In addition, all participants were at least overweight and may have type II diabetes. The analyzed studies used five different FGF21 analogs: 1) LY2405319, a human FGF21 analog containing modifications in its primary sequence to create greater stability and large-scale production (59); 2) PF-05231023, an analog consisting of two molecules of human FGF21 modified and conjugated to an antibody, aimed at increasing the half-life (60); 3) Pegbelfermin, a pegylated human FGF21 with an increased half-life (53); 4) AKR-001, human FGF21 with point mutations conjugated to an antibody, targeting greater receptor affinity and half-life (61); and 5) LLF580, human FGF21 stabilized by an inserted disulfide bond and antibody conjugation, increasing its half-life (57).

**Table S2:** Characteristics of the included studies and participants

Study	Sample size	Age (years)	Male (%)	White (%)	BMI (kg/m^2^)	Fasting glucose (mg/dL)	HbA1c (%)	Total cholesterol (mg/dL)	Systolic blood pressure (mmHg)	Fasting FFA (μmol/L)	Status of participants	Intervention
Gaich and cols., 2013	46	57.7	26 (57)	96	32.1	171.9	7.96	NA	NA	NA	Obesity and T2DM	Subcutaneous dose of LY2405319 (3, 10 or 20 mg) daily for 28 days
Dong and cols., 2015	84	55.5	60 (71)	92	30.4	173.4	8.60	NA	NA	NA	Overweight or obesity and T2DM	Single intravenous dose of PF-05231023 (0.5, 1.5, 5, 15, 50, 100 or 200 mg)
Talukdar and cols., 2016	50	55.7	39 (78)	90	29.7	168.0	8.18	NA	NA	NA	Overweight or obesity and T2DM	Intravenous dose of PF-05231023 (5, 25, 100 or 140 mg) twice weekly for four weeks
Kim and cols., 2017	107	53.4	76 (71)	89	34.0	NA	NA	153.8	121.6	NA	Obesity and hypertriglyceridemia (with or without T2DM)	Intravenous dose of PF-05231023 (25, 50, 100 or 150 mg) once weekly for four weeks
Charles and cols., 2019	96	56.0	53 (55)	76	35.0	151.0	7.80	NA	NA	NA	Obesity and T2DM	Subcutaneous dose of pegbelfermin (1, 5 or 20 mg daily, or 20 mg weekly) for 12 weeks
Kaufman and cols., 2020	69	55.5	39 (57)	NA	32.0	183.7	7.90	196.5	123.9	544.2	Overweight or obesity and T2DM	Subcutaneous dose of AKR-001 (7, 21, 70 or 140 mg) once weekly or once every two weeks twice for four weeks
Rader and cols., 2022	61	45.5	30 (49)	80	36.1	100.9	5.5	214.1	124.5	NA	Obesity and hypertriglyceridemia (with or without T2DM)	Subcutaneous dose of LLF580 (300 mg) every four weeks for 12 weeks
Loomba and cols., 2023	81	51.9	31 (38)	92	34.6	132.1	6.7	NA	NA	430.3	Overweight or obesity and NASH (with or without T2DM)	Subcutaneous dose of pegbelfermin (3, 9, 18 or 27 mg once weekly, or 18 or 36 mg once every two weeks) for 12 weeks

BMI: body mass index; FFA: free fatty acid; HbA1c: glycated hemoglobin; NA: not available; NASH: nonalcoholic steatohepatitis; T2DM: type II diabetes mellitus.

### Effects of FGF21 analogs on glucose homeostasis

**Figure 1:**
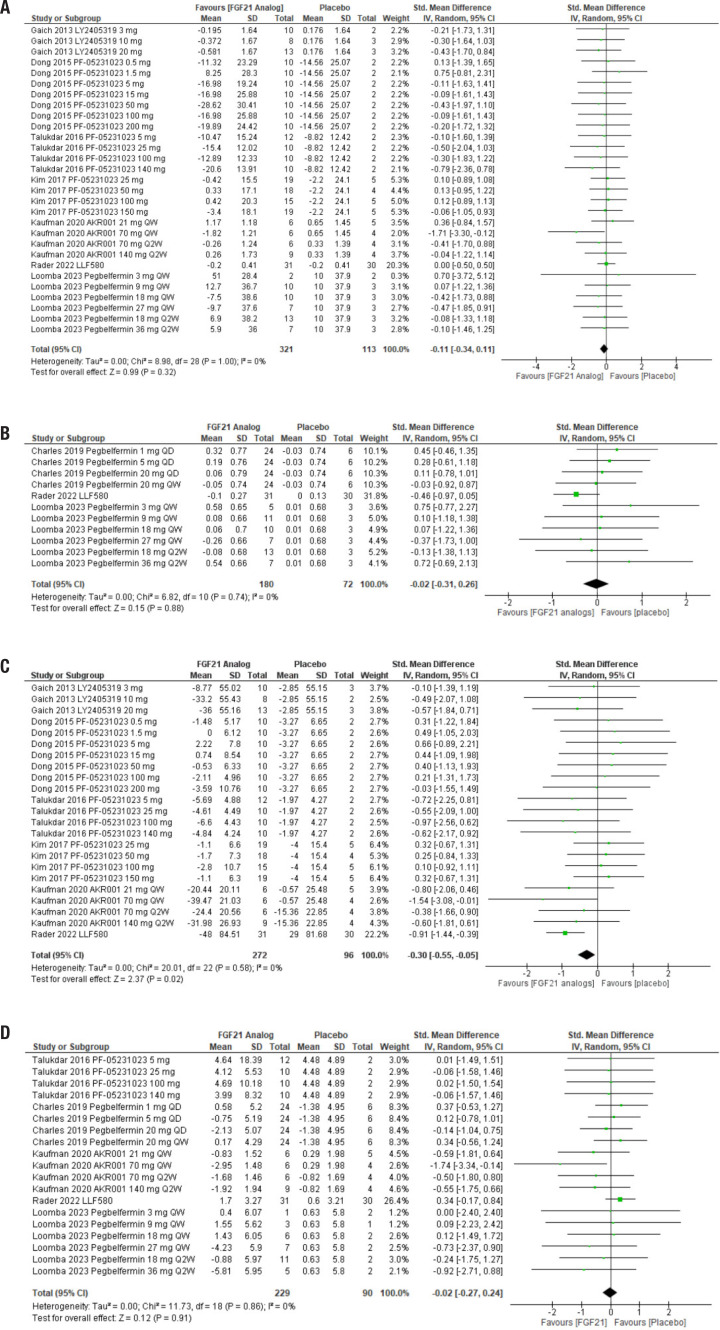
Forest plot comparing the effects of FGF21 analogs to placebo on glucose homeostasis and insulin resistance. The analog used and its dose are indicated in each line. QD: administered every day; QW: administered every week; Q2W: administered every two weeks. (**A**) Fasted glucose. (**B**) Glycated hemoglobin. (**C**) Fasted insulin. (**D**) HOMA index.

First, we analyzed the effect of FGF21 analogs on fasting blood glucose. This analysis included seven studies and 434 subjects, 321 in the treatment group and 113 in the control group. Treatment produced no effect on the outcome, with an estimated effect (95% CI) of −0.11 (−0.34, 0.11), Z = 0.99 (*p* = 0.32). The heterogeneity was I^2^ = 0% (Figure 1A).

Next, we evaluated glycated hemoglobin levels on FGF21 analog treatment in three studies with 252 participants, 180 in the treatment group and 72 in the placebo groups. Again, the use of FGF21 had no significant effect, with an estimated effect (95% CI) of −0.02 (−0.31, 0.26), Z = 0.15 (*p* = 0.88). As with the previous analysis, heterogeneity was low (Figure 1B).

Fasting insulinemia was significantly lower in participants who received treatment with FGF21 analogs, with an estimated effect (95% CI) of −0.30 (−0.55, −0.05), Z = 2.37 (*p* = 0.02). This analysis included 368 subjects, divided into 272 in the treatment groups and 96 in the control groups, for a total of six studies. Heterogeneity was also low (Figure 1C).

However, this reduction in fasting insulin did not reflect an improvement in the HOMA index. Treatment with FGF21 analogs had an estimated effect (95% CI) of −0.02 (−0.27, 0.24), Z = 0.12 (*p* = 0.91), in an analysis including five studies and 319 participants (229 in the treated groups and 90 in the control groups). Heterogeneity was low (Figure 1D).

### Effects of FGF21 analogs on body weight and blood pressure

**Figure 2:**
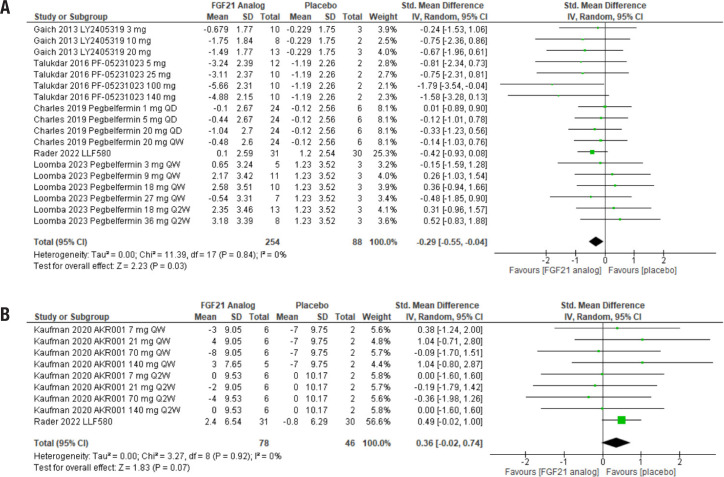
Forest plot comparing the effects of FGF21 analogs to placebo on body weight and systolic blood pressure. The analog used and its dose are indicated in each line. (**A**) Body weight. QD: administered every day; QW: administered every week. (**B**) Systolic blood pressure. QW: administered every week; Q2W: administered every two weeks.

Treatment with FGF21 analogs had a significant effect on participants’ body weight, with an estimated effect (95% CI) of −0.29 (−0.55, −0.04), Z = 2.23 (*p* = 0.03). The analysis included five studies, with 342 subjects, divided between 254 in the treatment groups and 88 in the control groups, and the heterogeneity was low (Figure 2A).

We evaluated the effect of FGF21 analogs on systolic blood pressure. The analysis included two studies and 124 participants, 78 in the treatment group and 46 in the control groups. The treatment did not change the outcome, with an estimated effect (95% CI) of 0.36 (−0.02, 0.74), Z = 1.83 (*p* = 0.07). As with other outcomes, heterogeneity was low (Figure 2B).

### Effects of FGF21 analogs on blood total cholesterol and free fatty acids

**Figure 3:**
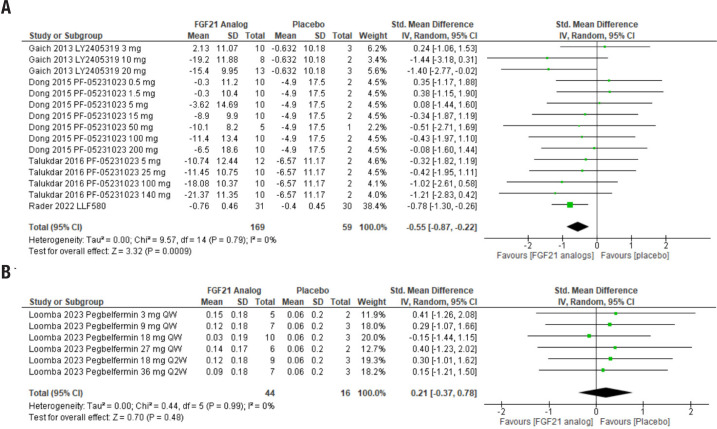
Forest plot comparing the effects of FGF21 analogs to placebo on total cholesterol and free fatty acids. The analog used and its dose are indicated in each line. (**A**) Total cholesterol. (**B**) Plasmatic free fatty acids. QW: administered every week; Q2W: administered every two weeks.

Total cholesterol levels were also much lower in the groups treated with FGF21 analogs. This treatment had an estimated effect (95% CI) of −0.55 (−0.87, −0.22), Z = 3.32 (*p* = 0.0009), in analysis with four studies and 228 participants, 169 in the treatment groups and 59 in the placebo groups. As with previous outcomes, heterogeneity was low (Figure 3A).

Finally, only one study verified the effects of treatment with FGF21 analogs on plasma levels of free fatty acids. The drug did not alter lipid levels, demonstrating an estimated effect (95% CI) of 0.21 (−0.37, 0.78), Z = 0.7 (*p* = 0.48). The analysis included 60 patients, 44 in the treatment group and 16 in the placebo group. The heterogeneity of the analysis was low.

### Analysis of risk of bias and quality of evidence

**Figure S2:**
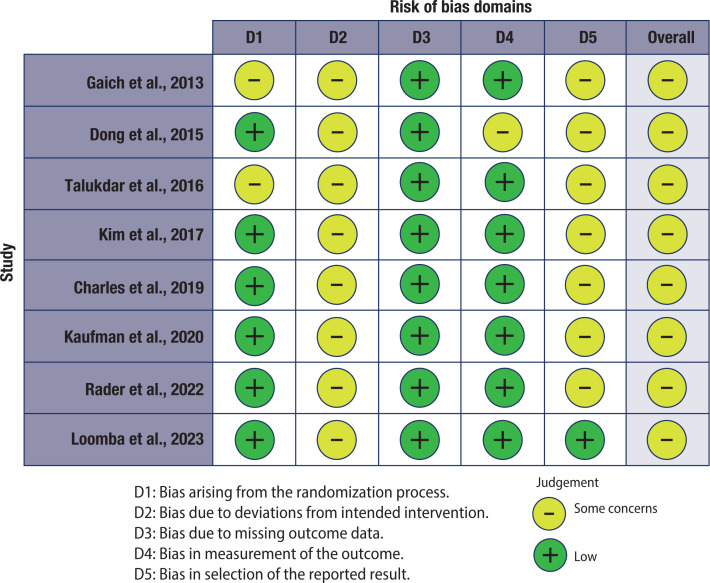
Bias risk analysis. The articles were analyzed according to the RoB 2 algorithm, and the figures were plotted with the RobVis web application.

We did not identify a high risk of bias in any of the studies included in this meta-analysis. However, in all studies, there are some concerns. According to our analysis of the articles, the lack of reported information does not allow us to exclude the risk of deviations in the intended interventions and the selection of reported results (Figure S2). However, we do not believe this dramatically impacts the quality of the evidence obtained.

**Figure S3:**
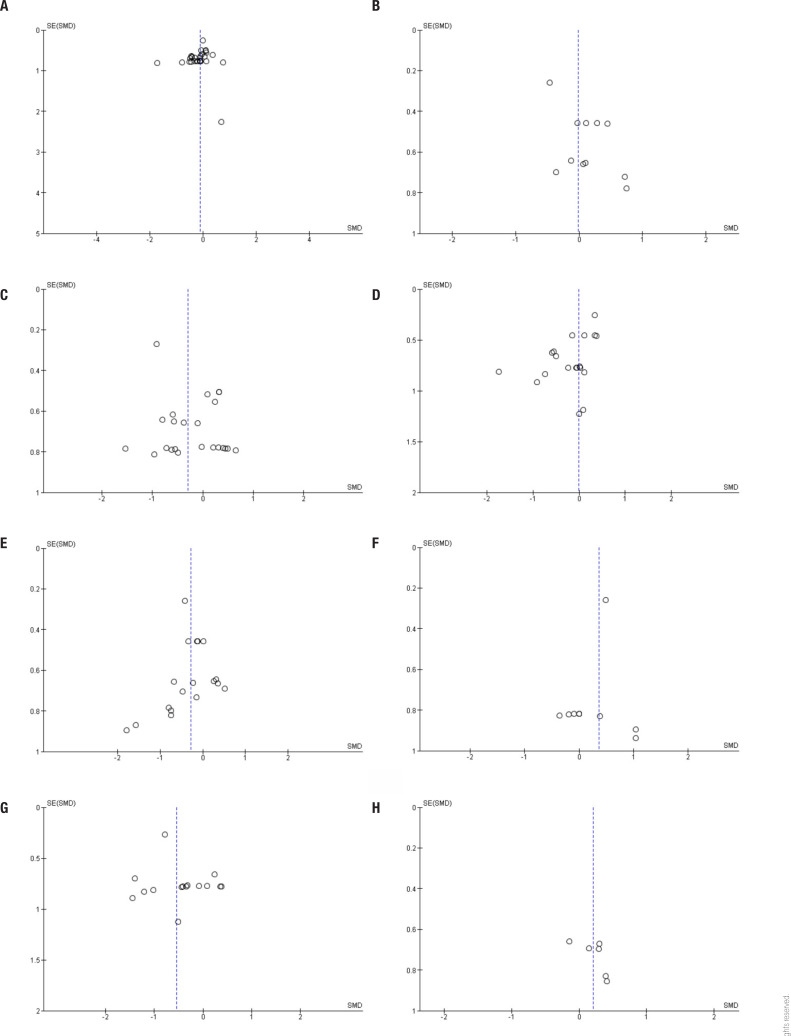
Funnel plots of comparison between FGF21 analogs and placebo. (**A**) Fasting glucose. Egger's test: p > 0.05. (**B**) Glycated hemoglobin. Egger's test: p < 0.05. (**C**) Fasting insulin. Egger's test: p < 0.05. (**D**) HOMA index. Egger's test: p < 0.05. (**E**) Body weight. Egger's test: p < 0.05. (**F**) Systolic blood pressure. Egger's test: p < 0.05. (**G**) Total cholesterol. Egger's test: p > 0.05. (**H**) Plasma free fatty acids. Egger's test: p < 0.05.

Table 1 summarizes the quality of evidence obtained in this meta-analysis. The quality of evidence ranges from moderate to very low. In almost all outcomes, we reduced quality due to indirectionality and imprecision. We also identified publication bias in glycated hemoglobin, fasting insulin, HOMA index, body weight, systolic blood pressure, and plasma free fatty acid outcomes (Figure S3). On the other hand, the sizeable estimated effect of the use of FGF21 analogs on cholesterolemia indicated an increase in the quality of the evidence.

**Table 1:** GRADE summary of findings

Outcomes	Number of participants (studies)	Quality of the evidence (GRADE)	Size effect (95% CI)
Fasting glucose	434 (7 studies)	Moderate, due to indirectness	−0.11 (−0.34 to 0.11)
Glycated hemoglobin	252 (3 studies)	Very low, due to indirectness, imprecision, and publication bias	−0.02 (−0.31 to 0.26)
Fasting insulin	368 (6 studies)	Very low, due to indirectness, imprecision, and publication bias	−0.30 (−0.55 to −0.05)
HOMA	319 (4 studies)	Very low, due to indirectness, imprecision, and publication bias	−0.02 (−0.27 to 0.24)
Body weight	342 (5 studies)	Very low, due to indirectness, imprecision, and publication bias	−0.29 (−0.55 to −0.04)
Systolic blood pressure	124 (2 studies)	Very low, due to indirectness, imprecision, and publication bias	0.36 (−0.02 to 0.74)
Total cholesterol	228 (4 studies)	Moderate, due to indirectness, imprecision, and size effect	−0.55 (−0.87 to −0.22)
Free fatty acids	60 (1 study)	Low, due to imprecision and publication bias	0.21 (−0.37 to 0.78)

## DISCUSSION

In this systematic review and meta-analysis, we evaluated the effects of FGF21 analogs as a treatment for metabolic disorders. Our searches allowed the inclusion of eight randomized clinical trials, and our assessments did not identify a high risk of bias in any of them. Three of the outcomes included in the analysis demonstrated significant results in the included studies, favoring the use of FGF21 analogs in the clinic: fasting blood insulin, body weight, and cholesterolemia.

Several preclinical trials have already reported on the effect of FGF21 on reducing plasma insulin levels. For example, FGF21 treatment in obese monkeys and mice reduced fasting insulinemia (25,31,33). Moreover, the administration of human FGF21 mRNA reduced insulinemia in mice with diet-induced obesity (21). Similarly, the FGF21 analog LY2405319 reduced fasting blood insulin in ApoE -/- mice on an atherogenic diet and insulin levels in diabetic monkeys (28,62). Gaich and cols. used this exact analog in 2013 in patients with obesity and diabetes, where the drug could also reduce insulin levels (56). Recombinant and PEGylated versions of human FGF21 also reduce insulinemia in obese mice and rats (30,37,40). Treatment with FGF21 receptor agonists has a similar effect in obese mice and monkeys (27,63). On the other hand, some studies indicate that using FGF21 may have the opposite effect and increase insulinemia in genetically obese *db*/*db* and type I diabetic mice (42,64,65). The reason for these differences is unclear but may involve using different analogs and treatment protocols.

Weight loss is also a commonly observed result in animals treated with FGF21. For example, using human FGF21 mRNA in obese mice reduces weight gain (21). FGF21 has a similar effect on obese mice, rats, and monkeys (9,25,27,29,31,33,37,42,63,66). Furthermore, different FGF21 analogs or FGF21 receptor agonists reduced the weight of obese models such as rats, monkeys, and mice (13,22,24). Again, the drug LY2405319 reduces the body weight of dyslipidemic mice, diabetic monkeys, and patients with obesity (28,56,62). Interestingly, FGF21 achieves this weight-reducing effect through an increase in energy expenditure (27,31), as the effects of this hormone on food consumption remains controversial (22,32,62).

Finally, preclinical trials also indicated that FGF21 has the potential to control cholesterolemia. Again, treating obese mice with human FGF21 mRNA reduced the animals’ cholesterolemia (21). Likewise, FGF21 or its analogs reduced plasma cholesterol levels in mice, rats, and monkeys (27,30,37,39,41,42). The LY2405319 analog demonstrated the same results in monkeys and patients with obesity (56,62). FGF21 likely reduces cholesterolemia through changes in the expression profile of genes involved in the metabolism of bile salts and cholesterol in the liver, in addition to reducing the capacity for hepatic cholesterol synthesis due to reduced activity of SREBP-2 (19,39).

However, our meta-analysis did not detect significant results in the other evaluated outcomes in the included studies: fasting glucose, glycated hemoglobin, HOMA index, blood free fatty acids, or blood pressure. Preclinical trials with FGF21 and its analogs commonly observe reduced blood glucose and improved glucose homeostasis in different models (13,21,23,27–34,37,40–43,62–67). Consequently, studies that investigated glycated hemoglobin levels and the HOMA index also reported beneficial effects after treatment with this hormone (25,28–30,37,42,64,65). FGF21 appears to act at different points to reduce plasma glucose levels. First, FGF21 increases glucose tolerance by inducing the expression of GLUT and hexokinase and glucose uptake by different tissues (13,23,27–29,31,33,34,37,42,43,63–65,67). In addition, this hormone stimulates glycogen synthesis in the liver and muscles (34,43). Finally, FGF21 reduces hepatic gluconeogenesis, decreasing the expression of enzymes in this pathway, such as G6Pase and PEPCK, which helps control blood glucose in diabetic models (27,33,34,42,64,65,68). However, glucose uptake by brown adipose tissue and increased energy expenditure via thermogenesis in this tissue appear to play an essential role in FGF21-mediated glycemic control (26,31,43). Brown adipose tissue activity in human subjects appears heterogeneous and inversely correlated with metabolic disturbances (69–71). These factors could help explain the lack of effect of FGF21 on blood glucose and related outcomes in the clinical studies included in this meta-analysis.

It is important to note that we detected a reduction in insulinemia and body weight with treatment with FGF21 analogs but without a change in the HOMA index. However, it is not easy to conclude mechanisms of metabolic regulation based on the results of a clinical meta-analysis because we could not estimate the absolute differences, only the estimated effect of treatment versus placebo. Thus, if the effect on insulinemia or weight is minimal, the effect on the HOMA index will be diluted and undetected. Finally, the quality of evidence for these outcomes still needs to be improved. Therefore, it is possible that the accuracy of the effect on insulinemia and weight needs to be refined, and future studies may demonstrate that the effect is insignificant.

Finally, our study indicated that using FGF21 analogs does not affect subjects’ blood pressure or plasma free fatty acid levels. Preclinical trials have explored these outcomes relatively little, but using the FGF21 analog PF-05231023 increased rat blood pressure (36). On the other hand, treatment of mice with FGF21 reduces plasma levels of free fatty acids (27,31). Differences in brown adipose tissue metabolism between mice and humans may also help explain the difference in the results obtained.

Overall, the quality of evidence obtained in this meta-analysis remains low. The low quality is primarily due to the small number of clinical studies performed. None of the outcomes included, except fasting glycemia, had a sample number greater than 400 individuals, which reduces the quality of the evidence due to the imprecision of the results. All studies included patients who were at least overweight and may have type 2 diabetes. In addition, two studies included patients with hypertriglyceridemia and one with nonalcoholic steatohepatitis. The homogeneous characteristics of the group of participants may have been responsible for keeping the heterogeneity of the meta-analysis results low in all outcomes. Therefore, we did not consider performing subanalyses with the meta-analysis data. However, to carry out the meta-analysis, we combined studies with different FGF21 analogs used at different doses. Therefore, we prefer to reduce the quality of evidence obtained due to indirectionality. Based on the quality of evidence obtained, we anticipate that more clinical trials using existing drugs could help increase the quality of evidence by increasing the number of participants, reducing imprecision, and reducing indirection by facilitating analyses with a single type of FGF21 analog.

In conclusion, FGF21 analogs must be tested in new clinical trials, as they appear to exhibit great potential for treating signs of Metabolic Syndrome such as high blood insulin, obesity, and especially hypercholesterolemia.
